# Results of video-assisted thoracoscopic surgery versus thoracotomy for lung cancer in a mixed practice medium-volume hospital: a propensity-matched study

**DOI:** 10.1093/icvts/ivad189

**Published:** 2023-11-27

**Authors:** Anne Niskakangas, Olli Mustonen, Iiris Puro, Topias Karjula, Olli Helminen, Fredrik Yannopoulos

**Affiliations:** Research Unit of Translational Medicine, Medical Research Center Oulu, University of Oulu, Oulu, Finland; Research Unit of Translational Medicine, Medical Research Center Oulu, University of Oulu, Oulu, Finland; Research Unit of Translational Medicine, Medical Research Center Oulu, University of Oulu, Oulu, Finland; Research Unit of Translational Medicine, Medical Research Center Oulu, University of Oulu, Oulu, Finland; Research Unit of Translational Medicine, Medical Research Center Oulu, University of Oulu, Oulu, Finland; Department of Gastrointestinal Surgery, Oulu University Hospital, Oulu, Finland; Research Unit of Translational Medicine, Medical Research Center Oulu, University of Oulu, Oulu, Finland; Department of Cardiothoracic Surgery, Oulu University Hospital, Oulu, Finland

**Keywords:** Lung cancer, Video-assisted thoracoscopic surgery, Thoracotomy, Survival, Mediastinal lymph node dissection, Nodal upstaging

## Abstract

**OBJECTIVES:**

The aim of this study was to compare the short- and long-term results of video-assisted thoracoscopic surgery (VATS) and thoracotomy for non-small-cell lung cancer in a medium-volume centre, where cardiothoracic surgeons perform both cardiac and general thoracic surgery. The primary outcome of interest was 5-year overall survival and disease-specific survival. Secondary outcomes were short-term postoperative complications, length of hospital stay and lymph node yield.

**METHODS:**

This was a retrospective cohort study including 670 lung cancer patients undergoing VATS (*n* = 207) or open surgery (*n* = 463) with a curative intent in Oulu University Hospital between the years 2000–2020. Propensity score matching was implemented with surgical technique as the dependent and age, sex, Charlson comorbidity index, pulmonary function, pathological stage, histological type and the year of the operation as covariates resulting in 127 pairs.

**RESULTS:**

In the propensity-matched cohort, 5-year overall survival was 64.3% after VATS and 63.2% after thoracotomy (*P* = 0.969). Five-year disease-specific survival was 71.6% vs 76.2% (*P* = 0.559). There were no differences in overall (34.6% vs 44.9%, p = 0.096) or major postoperative complications (8.7% vs 14.2%, *P* = 0.167) between the study groups. The average length of hospital stay was shorter (5.8 vs 6.6 days, *P* = 0.012) and the median lymph node yield was lower (4.0 vs 7.0, *P* < 0.001) in the VATS group compared to the thoracotomy group.

**CONCLUSIONS:**

According to this study, the long-term results of lung cancer surgery in a mixed practice are comparable between VATS and open surgery.

**Figure:**
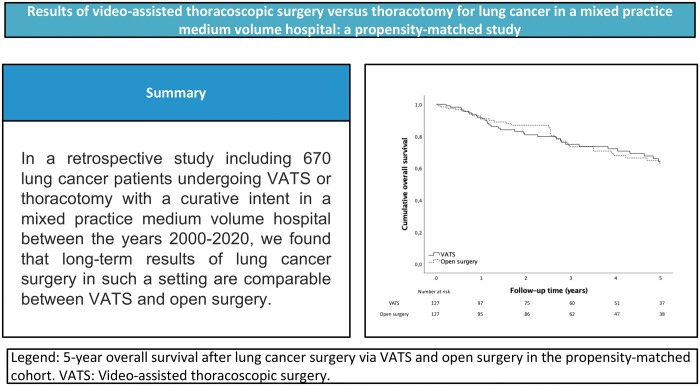

## INTRODUCTION

Lung cancer is the most common cause of cancer death worldwide [1]. In the early stages of the disease, surgical resection is the cornerstone of treatment [1, 2]. The most recent guidelines, European Society for Medical Oncology 2017 and American College of Chest Physicians 2013, recommend video-assisted thoracoscopic surgery (VATS) as the approach of choice in stage I non-small-cell lung cancer (NSCLC) over traditional thoracotomy [1, 2]. VATS lobectomy is associated with lower overall incidence of multiple postoperative complications, as well as a shorter hospital stay and chest tube duration [3, 4]. Despite its benefits, the adoption of VATS has been slow. European Society of Thoracic Surgery (ESTS) reports the rate of VATS operations in 2007–2022 as 42.2% of all lung resections [5]. In Finland, between 2010 and 2014 the rate of VATS was 32.9% with a clear increase in VATS usage during the decade [6].

From oncological perspective, results of VATS and open resection have been comparable [1]. The efficacy of VATS compared to open surgery concerning lymph node dissection is controversial [7–11]. Regardless, thoracoscopic approach to lung cancer surgery seems to result in at least equivalent long-term survival compared to open surgery [7, 12, 13]. Randomized trial and high-volume centre outcomes often differ from real-world practice. Cardiac and general thoracic surgery are often separated in high-volume centres, resulting in higher surgeon-specific volume, whereas in medium- or low-volume hospitals, same surgeons perform both cardiac and lung cancer surgery. To our knowledge, results of VATS compared to open surgery have not been reported in mixed practice.

The aim of the present study was to compare the results of VATS and open lung cancer surgery in a medium-volume centre, where cardiothoracic surgeons perform both cardiac and lung surgery. Primary outcomes of interest were 5-year overall survival (OS) and disease-specific survival (DSS). Secondary outcomes were short-term postoperative complications, length of hospital stay and lymph node yield.

## MATERIALS AND METHODS

### Study design

This was a retrospective cohort study with intention-to-treat-based inclusion of all lung cancer patients operated in Oulu University Hospital, Finland, between 1 January 2000 and 31 December 2020. Data are considered population-based, since during the period, nearly all elective lung cancer patients in Northern Finland were operated in Oulu University Hospital, which is the only university hospital in Northern Finland. The cohort size was 670 patients. The study was approved by the local hospital district and Northern Ostrobothnia Ethics Committee (EETTMK 81/2008).

### Inclusion criteria

All patients with histologically confirmed primary lung cancer operated with a curative intent were evaluated. For the final cohort, only NSCLCs were included. To maintain comparability between the VATS and thoracotomy groups, 174 patients who underwent pneumonectomy, sublobar resection or biopsy/enucleation were excluded since inclusion would have potentially skewed the results in favour of the VATS resection group.

### Data collection

The patients were retrospectively identified from electronic hospital records of Oulu University Hospital. Survival data and causes of death were obtained from the National Causes of Death Register with 100% coverage [14].

Patient and tumour information was collected from electronic patient charts and pathology reports by the authors. Tumour stage data were re-reviewed and updated according to the 8th edition of TNM Classification of Malignant Tumors [15].

### Outcomes and definitions

Main outcomes of interest were 5-year OS and DSS after lung cancer surgery. Secondary outcomes were short-term complications, hospital stay and lymph node yield.

Modified Charlson comorbidity index (CCI) was used to evaluate comorbidities and classified them as 0, 1, 2 or ≥3 [16]. Lung cancer under treatment was not counted as a comorbidity. Complications were classified using the Clavien–Dindo complication classification system [17]. Complications classified as Clavien–Dindo class III or more were considered major complications and Clavien–Dindo class I–II were considered minor complications. The extent of lymph node dissection was classified as no lymph node dissection, only N1-level dissection, limited N2-dissection meaning only 1–2 mediastinal lymph node stations were dissected or systematic N2-dissection meaning at least 3 mediastinal lymph node stations were dissected according to the ESTS 2006 guideline [18].

### Surgical technique

During the study period, the VATS technique used in the study hospital was multiportal, meaning that the procedure was performed via three or four 5- to 10-mm ports and a larger access incision without rib spreading. For thoracotomy, anterolateral or posterolateral incision was used. Conversions from VATS to open surgery were analysed in the VATS group as per intention-to-treat.

### Statistical methods

Mann–Whitney *U*-test and Pearson’s Chi-square were used to determine differences between study groups regarding patient characteristics, preoperative risk factors and short-term postoperative outcomes, as appropriate. Kaplan–Meier curves including a log-rank test were constructed to present 1-, 3- and 5-year survival separately for the study groups. Propensity scores were calculated via a logistic regression model with surgical technique as the dependent and age; sex; CCI (0, 1, 2, 3 or more); forced expiratory volume in 1 s (FEV1) (<60% and >60%) of reference; diffusing capacity for carbon monoxide (DLCO) (<60% and >60% of reference); pathological stage (I, II, III or IV); histological type (adenocarcinoma, squamous cell carcinoma or other NSCLC); and the year of the operation (divided as 5-year blocks) as covariates. Based on the score, 127 unique pairs between the groups were matched using a maximum of 0.02 difference in the predicted probability between pairs. For missing FEV1 and DLCO data, multiple imputation was performed with smoking as a predictor. If both FEV1 and DLCO values were missing, no imputation was performed. Statistical analyses were performed using IBM SPSS 26.0 (IBM Corp., Armonk, NY, USA). Analyses were exploratory in nature and confirmatory studies are deemed necessary.

## RESULTS

### Unmatched cohort

**Figure 1:**
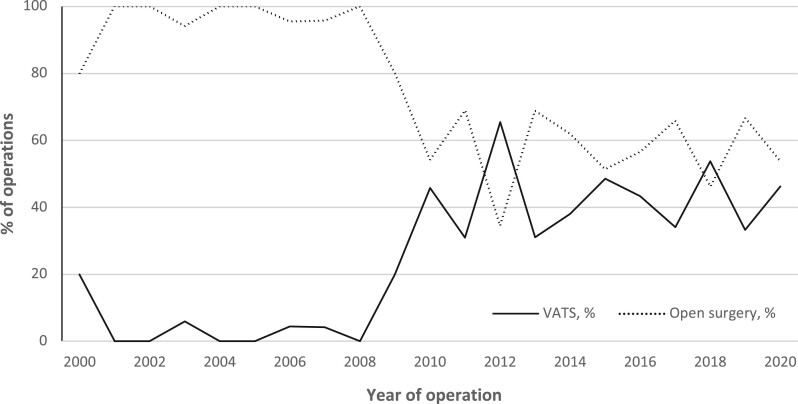
Trends in operative technique in 2000–2020 in Oulu University Hospital.

In the unmatched cohort, VATS was the approach of choice in 207 cases, whereas thoracotomy was used in 463 cases. There was an increase in the usage of VATS over the years: in 2000–2005, the rate of VATS was 3.1%, while in 2015–2020, it was 42.0% with a peak value of 65.5% in 2012 (Fig. 1). The conversion rate from VATS to open surgery was 23.7% during the study period. Conversions were made due to surgical reasons (51.0%), bleeding (22.4%), adhesions (16.3%), ventilation issues (8.1%) and problems with equipment (4.1%). Surgical reasons include conversions due to the lack of progression of surgery caused by, for example anatomical reasons. Between the VATS and open surgery groups, there were no statistically significant differences in patients’ age or comorbidity burden evaluated by CCI. The usage of positron emission tomography–computed tomography (PET-CT) increased over the years. Other NSCLCs included large cell lung carcinoma (*n* = 17), large cell neuroendocrine carcinoma (*n* = 10), carcinoid (*n* = 27), adenosquamous carcinoma (*n* = 9), sarcomatoid carcinoma (*n* = 15) and unclassified lung cancer (*n* = 1). For baseline characteristics of the unmatched cohort, see Table 1.

**Table 1:** Characteristics of patients who underwent surgery for lung cancer in Oulu University Hospital in 2000–2020, divided into subgroups by surgical technique

	Unmatched cohort			Matched cohort		
	VATS (*n* = 207)	Open surgery (*n* = 463)	*P*-value	VATS (*n* = 127)	Open surgery (*n* = 127)	*P*-value
Age (years), mean (SD)	68.2 (9.7)	67.3 (9.3)	0.224	67.5 (11.1)	67.5 (10.3)	0.831
Female gender, *n* (%)	89 (43.0)	135 (29.2)	<0.001	50 (39.4)	50 (39.4)	1.000
CCI, *n* (%)			0.582			0.883
CCI: 0	50 (24.2)	133 (28.7)		32 (25.2)	30 (23.6)	
CCI: 1	82 (39.6)	162 (35.0)		55 (43.3)	52 (40.9)	
CCI: 2	46 (22.2)	104 (22.5)		22 (17.3)	27 (21.3)	
CCI: 3+	29 (14.0)	63 (13.6)		18 (14.2)	18 (14.2)	
Smoking, *n* (%)			0.013			0.508
No	30 (14.5)	45 (9.7)		21 (16.5)	13 (10.2)	
Yes	75 (36.2)	215 (46.4)		51 (40.2)	56 (44.1)	
Ex-smoker	91 (44.0)	187 (40.4)		52 (40.9)	53 (41.7)	
Passive smoking	5 (2.4)	3 (0.6)		2 (1.6)	3 (2.4)	
Pulmonary function, *n* (%)						
FEV1 < 60%	22 (10.6)	92 (19.9)	0.003	16 (12.6)	20 (15.7)	0.472
FEV1 > 60%	182 (87.9)	361 (78.0)		111 (87.4)	107 (84.3)	
DLCO < 60%	33 (15.9)	78 (16.8)	0.742	23 (18.1)	24 (18.9)	0.872
DLCO > 60%	171 (82.6)	375 (81.0)		104 (81.9)	103 (81.1)	
Radiological staging, *n* (%)			<0.001			0.701
CT	93 (44.9)	285 (61.6)		65 (51.2)	67 (52.8)	
CT and PET–CT	114 (55.1)	173 (37.4)		62 (48.8)	58 (45.7)	
Neoadjuvant (yes), *n* (%)	2 (1.0)	15 (3.2)	0.084	2 (1.6)	2 (1.6)	1.000
Adjuvant, *n* (%)						
Yes	14 (6.8)	96 (20.7)	<0.001	12 (9.4)	14 (11.0)	0.578
No	151 (72.9)	270 (58.3)		91 (71.7)	84 (66.1)	
Operation type, *n* (%)			<0.001			0.432
Bilobectomy	16 (7.7)	59 (12.7)		12 (9.4)	14 (11.0)	
Lobectomy	173 (83.6)	378 (81.6)		107 (84.3)	104 (81.9)	
Segmentectomy	13 (6.3)	3 (0.6)		4 (3.1)	1 (0.8)	
Lobectomy and segmentectomy	0	2 (0.4)		0	1 (0.8)	
Lobectomy and sublobar resection	5 (2.4)	21 (4.5)		4 (3.1)	7 (5.5)	
Conversions, *n* (%)	49 (23.7)	0		32 (25.2)	0	
cStage, *n* (%)			<0.001			<0.001
0	0	1 (0.2)		0	1 (0.8)	
IA	156 (75.4)	137 (29.6)		92 (72.4)	51 (40.2)	
IB	22 (10.6)	73 (15.8)		13 (10.2)	25 (19.7)	
II	19 (9.2)	142 (30.7)		14 (11.0)	31 (24.4)	
III	8 (3.9)	96 (20.7)		6 (4.7)	16 (12.6)	
IV	0	1 (0.2)		0	0	
pStage, *n* (%)			<0.001			0.416
IA	142 (68.6)	141 (30.5)		75 (59.1)	71 (55.9)	
IB	28 (13.5)	44 (9.5)		18 (14.2)	24 (18.9)	
II	21 (10.1)	166 (35.9)		21 (16.5)	20 (15.7)	
III	14 (6.8)	109 (23.5)		13 (10.2)	12 (9.4)	
IV	0	3 (0.6)		0	0	
Histology, *n* (%)			<0.001			0.699
SCC	46 (22.2)	176 (38.0)		39 (30.7)	38 (29.9)	
Adenocarcinoma	141 (68.1)	228 (49.2)		71 (55.9)	76 (59.8)	
Other NSCLC	20 (9.7)	59 (12.7)		17 (13.4)	13 (10.2)	
Year of surgery, *n* (%)			<0.001			0.668
2000–2005	3 (1.4)	93 (20.1)		2 (1.6)	5 (3.9)	
2006–2010	19 (9.2)	107 (23.1)		19 (15.0)	16 (12.6)	
2011–2015	78 (37.7)	110 (23.8)		51 (40.2)	52 (40.9)	
2016–2020	107 (51.7)	153 (33.0)		55 (43.3)	54 (42.5)	

CCI: Charlson comorbidity index; CT: Computed tomography; DLCO: diffusing capacity for carbon monoxide; FEV1: forced expiratory volume in 1 s; NSCLC: non-small-cell lung cancer; SCC: squamous cell carcinoma; SD: standard deviation; PET-CT: Positron emission tomography–computed tomography; VATS: video-assisted thoracoscopic surgery.

Five-year OS was 53.3% and 5-year DSS was 64.9% for the unmatched cohort. Three-year OS was 65.8% and DSS 73.0%. One-year OS was 88.1% whereas DSS was 90.3%. The median follow-up time was 2.7 [interquartile range (IQR) 2.4–3.0] years.

#### Time-trend evolution

In the unmatched cohort, the rate of conversions did not change significantly over the study period (*P* = 0.308). In the VATS group, the lymph node yield remained similar over the years (*P* = 0.701), whereas in the thoracotomy group, it increased from median 3.0 (IQR 2–6) in 2000–2005 to 8 (IQR 5–14) in 2016–2020 (*P* < 0.001). The rate of overall complications did not change significantly over the study period (VATS *P* = 0.471, thoracotomy *P* = 0.350). Length of hospital stay remained similar in the VATS group (*P* = 0.215) but decreased in the thoracotomy group by median of 2 days over the years (*P* < 0.001). For the results on time-trend evolution, see Supplementary Material, Table S1 and Supplementary Material, Fig. S1.

### Propensity score-matched cohort

After propensity score matching, no statistically significant differences were observed between VATS and open surgery groups in covariates selected for propensity scoring (Table 1).

#### Survival

**Figure 2:**
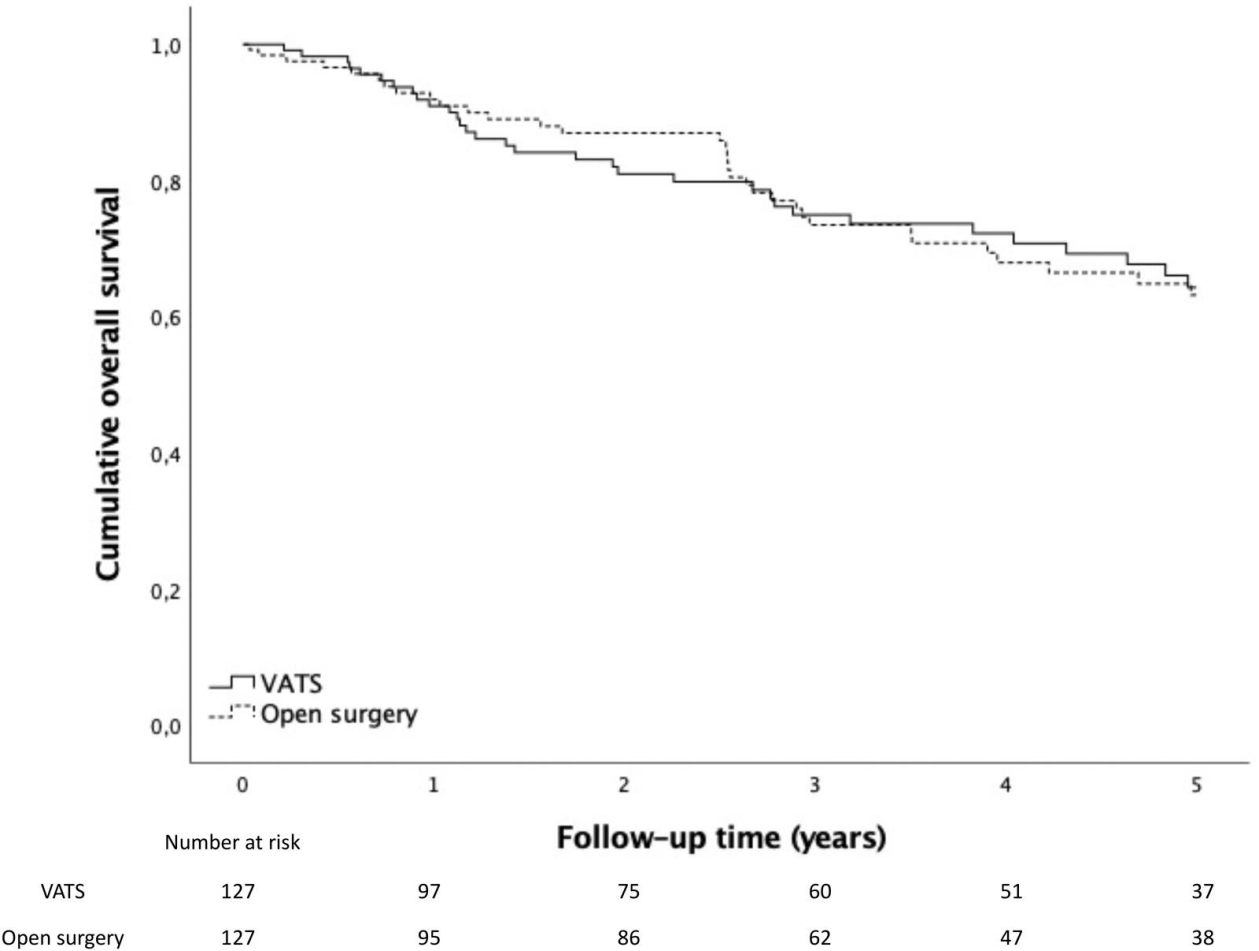
Five-year overall survival after lung cancer surgery via VATS and open resection in the propensity-matched cohort. VATS: video-assisted thoracoscopic surgery.

**Figure 3:**
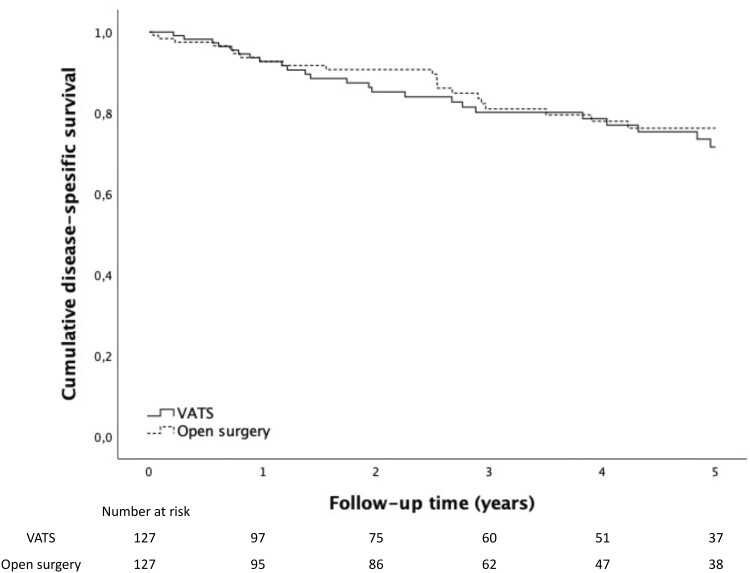
Five-year disease-specific survival after lung cancer surgery via VATS and open resection in the propensity-matched cohort. VATS: video-assisted thoracoscopic surgery.

Results in 5-year OS after lung cancer surgery were similar between the 2 groups in the propensity-matched cohort: 64.3% in the VATS group and 63.2% in the open surgery group (*P* = 0.969). Five-year DSS was 71.6% in the VATS group and 76.2% in the open surgery group (*P* = 0.559). Three-year OS was 74.9% vs 73.5% and DSS 80.1% vs 81.0%. One-year OS was 90.9% and 91.9% and DSS 92.7% in both groups. Survival curves are presented in Figs 2 and 3. We also performed survival analysis stratified according to pathological stage in the propensity-matched cohort—no significant difference could be demonstrated in 5-year OS or DSS between the groups. For the stratified analysis, see Supplementary Material, Table S2 and Supplementary Material, Fig. S2.

#### Postoperative outcomes

In the propensity-matched cohort, overall rate of postoperative complications in VATS and open groups were 34.6% and 44.9% (*P* = 0.096) and major complications 8.7% and 14.2% (*P* = 0.167). Conversion rate in the VATS group was 25.2%. After VATS, the mean length of hospital stay was shorter than after open surgery (5.8 vs 6.6 days, *P* = 0.012). Some 4.0% of VATS and 10.3% of open surgery patients spent at least 1 day in the intensive care unit (ICU) (*P* = 0.047). In the VATS group, more patients were discharged directly to home compared to the open surgery group (56.7% vs 42.5%, *P* = 0.041). Difference in the amount of reoperations was not statistically significant between the groups (3.2% after VATS vs 6.3% after thoracotomy, *P* = 0.497). Radical resection was reached in 94.5% of VATS operations and in 93.7% of thoracotomies (*P* = 0.214). There was no difference in 30-day mortality (VATS 0% vs thoracotomy 1.6%, *P* = 0.156) and 90-day mortality (0.8% vs 2.4%, *P* = 0.313) between the groups. Postoperative outcomes are presented in Table 2.

**Table 2:** Postoperative outcomes in the propensity-matched cohort

	VATS (*n* = 127)	Open surgery (*n* = 127)	*P*-value
Overall complications, *n* (%)	44 (34.6)	57 (44.9)	0.096
Minor complications, *n* (%)	33 (26.0)	39 (30.7)	0.404
Major complications, *n* (%)	11 (8.7)	18 (14.2)	0.167
Length of hospital stay (days)			0.012
Median (IQR)	5.0 (3.0–6.0)	5.0 (4.0–7.0)	
Mean (SD)	5.8 (4.6)	6.6 (4.2)	
Discharge to, *n* (%)			0.041
Home	72 (56.7)	54 (42.5)	
Other facility	55 (43.3)	70 (55.1)	
Reoperation, *n* (%)			0.497
During hospital stay	3 (2.4)	6 (4.7)	
Later	1 (0.8)	2 (1.6)	
Radical resection rate, *n* (%)	120 (94.5)	119 (93.7)	0.214
30-Day mortality, *n* (%)	0	2 (1.6)	0.156
90-Day mortality, *n* (%)	1 (0.8)	3 (2.4)	0.313
Site of recurrence, *n* (%)			0.938
Local, operated area	2 (1.6)	3 (2.4)	
Local, same lung	7 (5.5)	7 (5.5)	
Local, mediastinum	4 (3.1)	7 (5.5)	
Systemic	18 (14.2)	18 (14.2)	
Missing	29 (22.8)	20 (15.7)	

IQR: interquartile range; SD: standard deviation; VATS: video-assisted thoracoscopic surgery.

#### Lymph node dissection and upstaging

In the propensity-matched cohort, the median lymph node yield was lower in the VATS group compared to the open surgery group [4 (IQR 2–8) vs 7 (IQR 4–12), *P* < 0.001]. Lymph node dissection was less extensive in the VATS group (*P* < 0.001); in 16.5% of operations via VATS systematic N2-station dissection was performed, while via thoracotomy the rate of systematic N2-dissection was 37.0%. The total number of lymph node stations dissected was lower in the VATS group [median 2 (IQR 1–4) vs 3 (IQR 2–4), *P* < 0.001]. However, there was more nodal upstaging in the VATS group compared to the thoracotomy group in the matched cohort (14.2% vs 8.7%), but the difference between the groups was not statistically significant (*P* = 0.174). Outcomes regarding lymph node dissection and upstaging are presented in Table 3.

**Table 3:** Lymph node sampling and upstaging in the propensity-matched cohort

	VATS (*n* = 127)	Open surgery (*n* = 127)	*P*-value
Lymph node yield (*n*), median (IQR)	4 (2–8)	7 (4–12)	<0.001
Lymph node sampling, *n* (%)			<0.001
No sampling	23 (18.1)	7 (5.5)	
N1 sampling	24 (18.9)	17 (13.4)	
Limited (1–2 N2 stations)	59 (46.5)	56 (44.1)	
Systematic (≥3 N2 stations)	21 (16.5)	47 (37.0)	
Number of lymph node stations sampled, median (IQR)	2 (1–4)	3 (2–4)	<0.001
Total upstaging, *n* (%)	40 (31.5)	20 (15.7)	0.003
Nodal upstaging, *n* (%)	18 (14.2)	11 (8.7)	0.174

IQR: interquartile range; VATS: video-assisted thoracoscopic surgery.

#### Per protocol subgroup analysis

To compare the short-term outcomes after conversion from VATS to thoracotomy to those after a planned thoracotomy, the VATS group was further split into converted (*n* = 32) and unconverted (*n* = 95) subgroups. Overall rate of postoperative complications in the converted group was 56.3%, whereas in the planned thoracotomy group, it was 44.9% (*P* = 0.250). The rate of major complications was 18.8% vs 14.2% (*P* = 0.518), respectively. The median length of hospital stay after a converted procedure was 6 (IQR 4–6) days, whereas after a planned thoracotomy, it was 5 (IQR 3–6) days (*P* = 0.373). Thirty-day mortality was 0% for the converted group and 1.6% for the planned thoracotomy group (*P* = 0.475) and 90-day mortality was 0% vs 1.9% (*P* = 0.380).

## DISCUSSION

The main finding of this retrospective cohort study from a single centre in northern Finland was that there were no significant differences in OS or DSS between the VATS and thoracotomy groups in any of the time spans inspected up to 5 years after propensity score matching. This is in line with previous findings; multiple studies have shown the thoracoscopic approach to result in at least equivalent, if not better, long-term survival compared to open surgery [7, 12, 13, 19–21]. The findings of a few recent studies even suggest towards an improved 5-year survival after lung cancer surgery via VATS [22–24]. In our cohort, the rate of VATS usage was 42% of lung cancer operations between the years 2015–2020, with a peak value of 65.5% in 2012. This is in line with the Finnish national rate of 44.4% in 2014 and the European rate of 42.2% in 2007–2022 [5, 6].

Lung cancer surgery via VATS is associated with a lower incidence of postoperative complications and shorter hospital stay compared to open surgery [3, 4]. In our propensity-matched cohort, the numbers of overall, minor and major complications were slightly lower in the VATS group compared to the open surgery group, although without statistical significance. Hospital stay was slightly shorter after VATS, mean 0.8 days between the groups, which is not as prominent as in the ESTS database, where the average difference in length of hospital stay was 2 days between the approaches [3]. The reason behind this difference to the ESTS database could be the high conversion rate in our cohort, as well as the more frequent discharge to a rehabilitation centre instead of home in the open surgery group.

The possible incompleteness of mediastinal lymph node assessment has been at the core of concern when it comes to the oncologic results of VATS for lung cancer [1, 25]. Some single-centre studies have found the VATS approach to yield less lymph nodes than thoracotomy, and lower upstaging after VATS versus thoracotomy has been noted without effect on survival [8, 26, 27]. Our study supports these findings. We found a statistically significant difference in lymph node yield and the number of lymph node stations sampled between VATS and thoracotomy in favour of the open approach. As well, systematic lymph node dissection was performed more often via thoracotomy. However, the nodal upstaging rate was slightly higher in the VATS group but the difference was not statistically significant. This is most likely due to propensity score matching: nodal upstaging leads to a higher overall pathological stage, which resulted in patients operated via VATS with nodal upstaging to match better with patients operated via thoracotomy, who as a group had a higher pathological stage to begin with. Thus, all VATS patients with nodal upstaging in our original cohort got included in the propensity-matched cohort. This hypothesis is supported by the notion of higher nodal upstaging in the thoracotomy group in the original cohort. It is also the most likely explanation to the statistically significant difference in total upstaging in the propensity score-matched cohort.

One of the strengths of our study is the access to all patient records, providing a wider coverage of data than most registries. The identity number system and compulsory registries of Finland enabled definite identification of patients and obtainment of complete survival and cause of death data with 100% coverage. We were also able to obtain data of known preoperative prognostic factors, allowing us to perform propensity score matching to minimize bias in the comparison. The single-centre design of this study provides some advantages. The operations were performed by a small group of cardiothoracic surgeons in the same hospital with very little variation within the study period, possibly enhancing homogeneity in surgical practice and perioperative treatment to make the comparison more reliable. The fact that the surgeons operating on lung cancer in the study hospital perform both cardiac and general thoracic surgery is also noteworthy—a lot of studies on VATS versus thoracotomy are based on data from high-volume centres, where cardiac and general thoracic surgery are often separated, resulting in a high surgeon-specific volume of lung resections. No studies specifying a mixed practice setting regarding this kind of research question were found, although studies on the impact of hospital and surgeon volume on lung cancer treatment outcomes have been made [28, 29].

The weaknesses of our study include the rather small number of patients despite the 20-year timespan. Due to the retrospective study design, some information was missing. Especially for patients from different hospital districts, follow-up information regarding short-term complications was sometimes missing. Therefore, it is possible that some minor complications went unreported. In addition, we were unable to obtain data on some postoperative outcomes such as level of pain and chest tube duration as they were not consistently documented. When comparing 2 surgical approaches, the high conversion rate could be considered a weakness as well. It seems that in our cohort the threshold to perform conversion is quite low, but as our subgroup analysis demonstrates, the conversion does not seem to compromise short-term results compared to a planned thoracotomy.

## CONCLUSION

In a mixed cardiothoracic practice, VATS and open surgery resulted in similar long-term OS and DSS. Additionally, the rate of short-term complications between the 2 approaches was found to be comparable.

## Supplementary Material

ivad189_Supplementary_Data

## Data Availability

The data behind this article are available on a reasonable request to corresponding author. Before data sharing, a new ethics committee statement is needed.

## Notes

Not presented at a meeting or submitted to any future meeting.

## ABBREVIATIONS

CCI: Charlson comorbidity index
CT: Computed tomography
DLCO: Diffusing capacity for carbon monoxide
DSS: Disease-specific survival
ESTS: European Society of Thoracic Surgery
FEV1: Forced expiratory volume in 1 s
ICU: Intensive care unit
IQR: Interquartile range
NSCLC: Non-small-cell lung cancer
OS: Overall survival
PET-CT: Positron emission tomography–computed tomography
VATS: Video-assisted thoracoscopic surgery
