# Supplementary material for: Results of video-assisted thoracoscopic surgery versus thoracotomy for lung cancer in a mixed practice medium-volume hospital: a propensity-matched study
Source: Interdiscip Cardiovasc Thorac Surg. 2023 Nov 27;37(6):ivad189. doi: 10.1093/icvts/ivad189 (PMC10701201; doi:10.1093/icvts/ivad189)
Supplement: ivad189_Supplementary_Data [file ivad189_supplementary_data.zip › VATSvsThoracotomy_supplementarydata.docx]

**SUPPLEMENTARY MATERIAL**

**Table S1.** Main short-term outcomes divided in groups by year of operation. Unmatched cohort.

| Year of operation* |  | 2000-2005 | 2006-2010 | 2011-2015 | 2016-2020 | p-value |
| --- | --- | --- | --- | --- | --- | --- |
| Conversions, n (%) | VATS | 2 (66.7) | 5 (26.3) | 16 (20.5) | 26 (24.3) | 0.308 |
| Lymph node yield (n), median (IQR) | VATS | 3.0 (1-NA) | 3.0 (1-9) | 4.0 (2-8) | 5.0 (2-8) | 0.701 |
|  | Open surgery | 3.0 (2-6) | 6.0 (4-11) | 9.0 (5-14) | 8.0 (5-14) | <0.001 |
| Overall complications, n (%) | VATS | 1 (33.3) | 8 (42.1) | 22 (28.2) | 42 (39.3) | 0.417 |
|  | Open surgery | 41 (44.1) | 53 (49.5) | 59 (53.6) | 66 (43.4) | 0.350 |
| Length of hospital stay (days), median (IQR) | VATS | 6.0 (6-6) | 5.0 (5-6) | 5.0 (3-6) | 4.0 (3-7) | 0.215 |
|  | Open surgery | 7.0 (5-11) | 7.0 (5-8) | 5.0 (4-8) | 5.0 (4-6) | <0.001 |

*2000-2005: n(VATS)=3, n(open surgery)=93

2006-2010: n(VATS)=19, n(open surgery)=107

2011-2015: n(VATS)=78, n(open surgery)=110

2016-2020: n(VATS)=107, n(open surgery)=153

Table S2. 5-year survival after lung cancer surgery via VATS and open surgery stratified according to pathological stage in the propensity-matched cohort.

| pStage | OS VATS (%) | OS Thoracotomy (%) | p-value | DSS VATS (%) | DSS Thoracotomy (%) | p-value |
| --- | --- | --- | --- | --- | --- | --- |
| IA | 70.5 | 66.0 | 0.605 | 73.6 | 82.4 | 0.396 |
| IB | 64.3 | 72.5 | 0.402 | 80.4 | 81.6 | 0.727 |
| II | 66.9 | 52.0 | 0.790 | 78.1 | 52.0 | 0.324 |
| III | 30.9 | 41.1 | 0.938 | 33.8 | 60.0 | 0.601 |

Figure legends for supplementary material

Figure S1. Total lymph node yield via VATS and open resection in lung cancer operations by year in 2000-2020. Unmatched cohort.

Figure S2. Kaplan-Meier curves of 5-year overall survival after lung cancer surgery via VATS and open surgery stratified according to pathological stage in the propensity-matched cohort. A: pStage IA; B: pStage IB; C: pStage II; D: pStage III.
