# Supplementary figures and images for: Results of video-assisted thoracoscopic surgery versus thoracotomy for lung cancer in a mixed practice medium-volume hospital: a propensity-matched study
Source: Interdiscip Cardiovasc Thorac Surg. 2023 Nov 27;37(6):ivad189. doi: 10.1093/icvts/ivad189 (PMC10701201; doi:10.1093/icvts/ivad189)

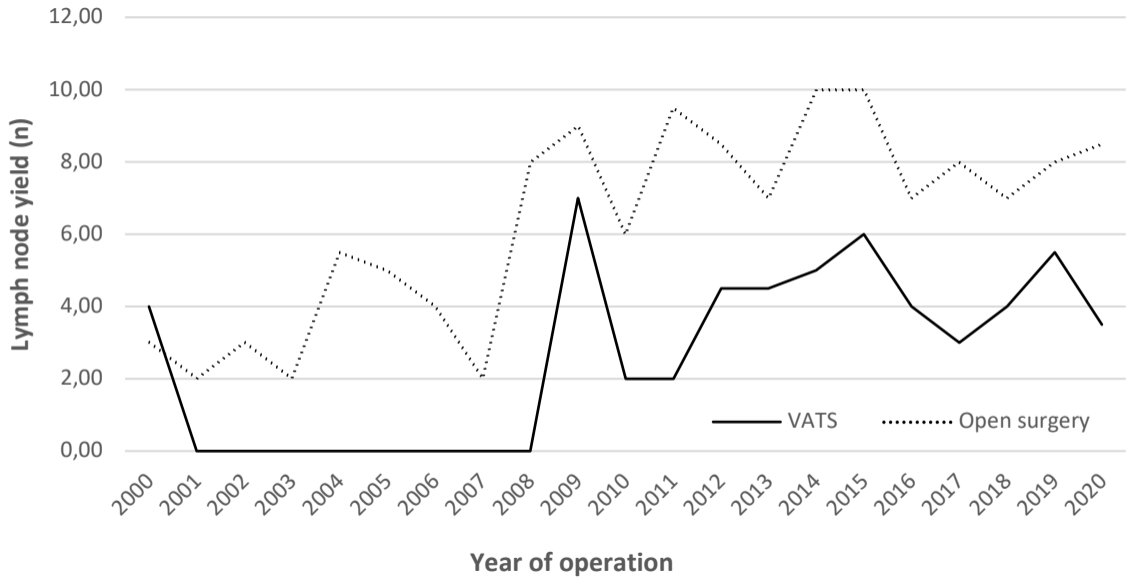

Supplement: ivad189_Supplementary_Data [file ivad189_supplementary_data.zip › FigS1.pdf]

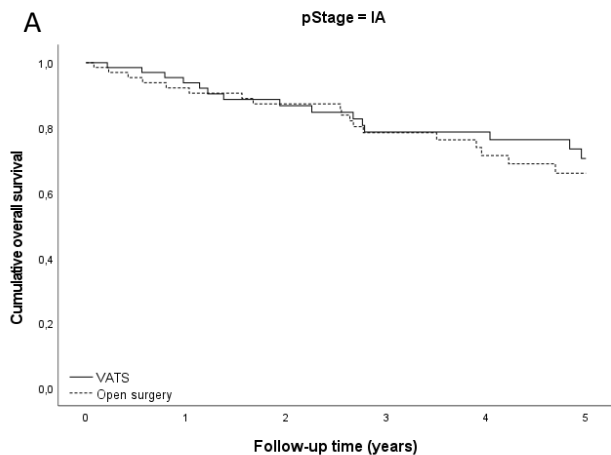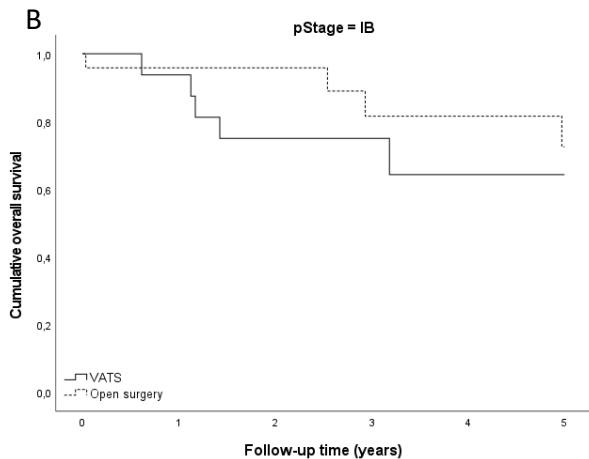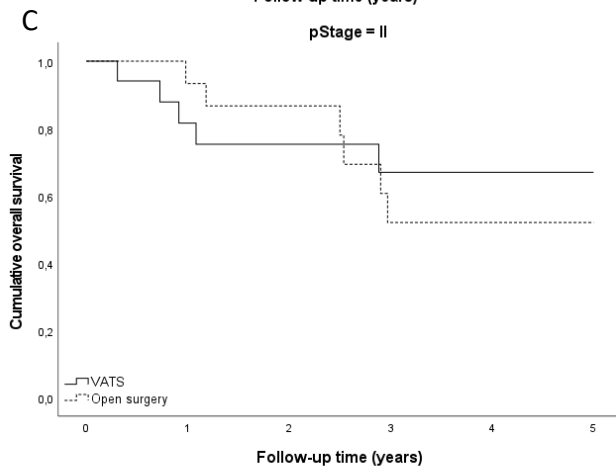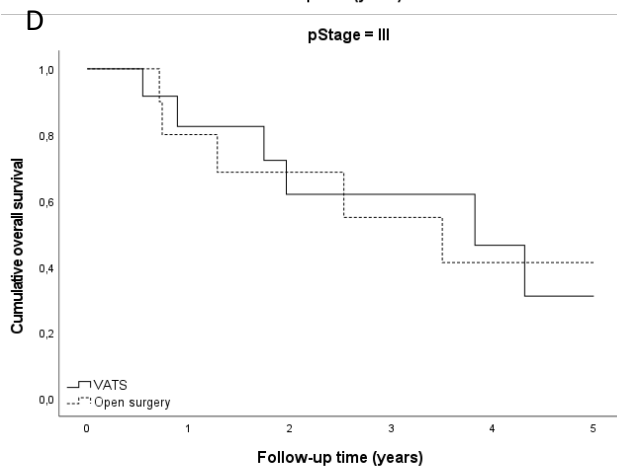

Supplement: ivad189_Supplementary_Data [file ivad189_supplementary_data.zip › FigS2.pdf]
